# Morphologic and Clinical Outcome of Intracranial Aneurysms after Treatment Using Flow Diverter Devices: Mid-Term Follow-Up

**DOI:** 10.1155/2016/2187275

**Published:** 2016-02-23

**Authors:** Anna-Katharina Breu, Till-Karsten Hauser, Florian H. Ebner, Felix Bischof, Ulrike Ernemann, Achim Seeger

**Affiliations:** ^1^Department of Diagnostic and Interventional Neuroradiology, Eberhard Karls University, Hoppe-Seyler-Street 3, 72076 Tübingen, Germany; ^2^Department of Neurosurgery, Eberhard Karls University, Hoppe-Seyler-Street 3, 72076 Tübingen, Germany; ^3^Department of Neurology, Eberhard Karls University, Hoppe-Seyler-Street 3, 72076 Tübingen, Germany

## Abstract

Flow diverters (FDs) are designed for the endovascular treatment of complex intracranial aneurysm configurations. From February 2009 to March 2013 28 patients (22 females, 6 males) were treated with FD; mean age was 57 years. Data, including aneurysm features, clinical presentation, history of previous bleeding, treatment, and follow-up results, are presented. Early postinterventional neurological deficits (transient: *n* = 3/enduring: *n* = 1) appeared in 4/28 patients (14%), and early improvement of neurological symptoms was observed in 7 patients with previous restriction of cranial nerve function. The overall occlusion rate was 20/26 (77%; 59% after 3 months). 77% achieved best results according to O'Kelly-Marotta score grade D with no contrast material filling (70% of those after 3 months). In 4/6 patients who did not achieve grade D, proximal and/or distal stent overlapping ≥5 mm was not guaranteed sufficiently. During follow-up we did not detect any aneurysm recurrence or haemorrhage. In-stent stenosis emerged as the most frequent complication (4/27; 15%) followed by 2 cases of vascular obliteration (AICA/VA). In conclusion endovascular reconstruction using a FD represents a modern and effective treatment in those aneurysms that are not suitable for conventional interventional or surgical treatment. The appearance of severe complications was rare.

## 1. Introduction

Low porosity stents (flow diverter, FD) play an important role in the effective endovascular treatment of intracranial aneurysms [1–3]. This device is designed for complex aneurysm configurations, fusiform, or wide-necked aneurysms, especially in cases where conventional coiling is not feasible and in locations where clipping is not a treatment option. Even remnants of aneurysms after surgical or endovascular treatment and dissected vessels in selected cases represent an indication for flow diverting treatment [4, 5]. The FD features two main work mechanisms: flow redirection and tissue overgrowth. The high-structural-profile implant bridges the aneurysm neck and reduces the blood flow into the aneurysm sac because of increased impedance created by the mesh of the implant with high pore density, yet providing blood flow through adjacent perforators and side branches. Flow stasis and formation of a stable aneurysmal thrombus are promoted by reduction of blood circulation within the aneurysm. In addition the FD provides a scaffold for neoendothelialization across the aneurysm neck, which leads to the exclusion of the aneurysm sac from the blood flow in the parent artery and facilitates a sufficient aneurysm occlusion [6]. Indeed questions remain regarding the long-term safety, radiological findings, and clinical outcome. The Flow Diversion in Intracranial Aneurysm Treatment (FIAT) [7] and the Large Aneurysm Randomized Trial: Flow Diversion Versus Traditional GDC Based Endovascular Therapy (LARGE) [8] are two of the ongoing randomized trials comparing flow diversion with best standard treatment. Interest in the future of flow diversion seems to be rapidly growing. Regarding aneurysm occlusion and vessel remodeling, the early follow-up results are encouraging, but long-term data are needed to assess efficacy and safety. This paper presents a systematic retrospective analysis of the technical findings concerning FD treatment as well as occlusion rate and clinical outcome in mid-term follow-up from 3 months to 3 years.

## 2. Materials and Methods

From February 2009 to November 2013, 28 patients (22 females, 6 males) were treated with FD; median age was 57 years (range: 8–83 years). A supplementary table providing all patients' clinical symptoms, aneurysm size, and location as well as treatment effects is provided (see supplementary materials in Supplementary Material available online at http://dx.doi.org/10.1155/2016/2187275). Clinical and radiological data were collected retrospectively and the maximum time of follow-up was 3 years.

### 2.1. Clinical Data

We treated 7 of 28 patients (25%) with FD who had ruptured aneurysms with previous SAH (Hunt & Hess II = 2, III = 1, V = 2; two without specification); 2/7 patients were treated in the acute period after SAH. We retreated one patient, who had a delayed aneurysm rupture 3 months after FD implantation in another centre due to insufficient aneurysm thrombosis.

**Figure 1:**
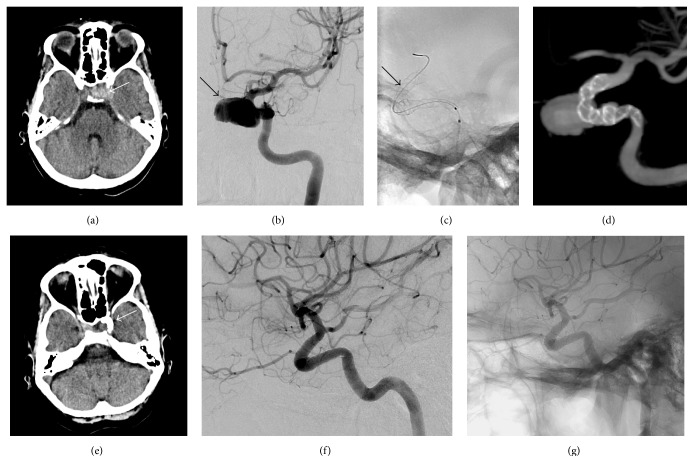
Cranial nerve palsy (patient number 1). 55-year-old woman presented with left oculomotor palsy. Cranial CT (a) showed hyperdense mass in the ophthalmic segment of the left carotid artery. Wide-necked intracranial aneurysm was proved by digital subtraction angiography (DSA, b) and the patient was treated using a flow diverter (c, d). In follow-up, clinical symptoms improved; the mass (e) and DSA confirmed no filling of the aneurysm sack (f, g).

**Figure 2:**
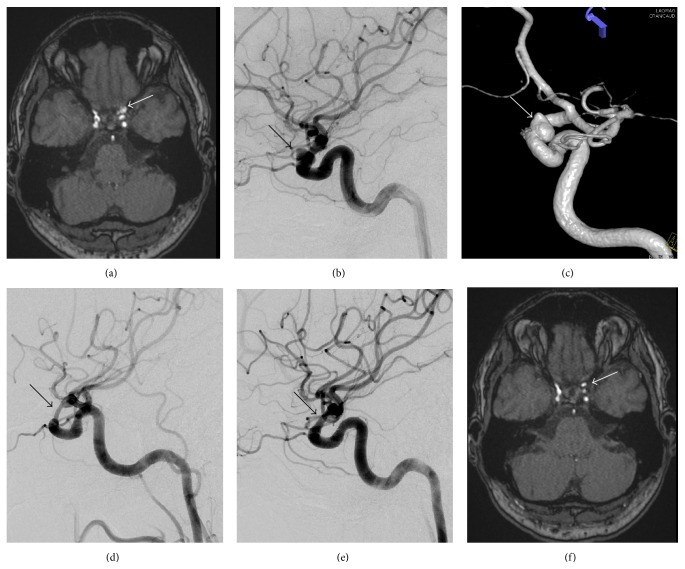
Postinterventional stenosis (patient number 25). 44-year-old man with incidental finding of aneurysm in the left carotid artery on MR angiography (a), confirmed by digital subtraction angiography (b, c). After flow diverter treatment, nonsymptomatic stenosis was detected and was stable in 6 months' follow-up (d); stenosis improved at 12 months' follow-up (e) and further noninvasive examinations.

12/28 patients (43%) had cranial nerve palsy due to compression by the aneurysm (e.g., patient number 1, see Figure 1). Five patients had unilateral visual loss, four (14%) had affection of the oculomotor nerve, one patient (4%) had affection of the abducens nerve, and two patients (7%) had affection of the fifth and ninth cranial nerve. One patient (4%) had four episodes of transient ischemic attacks. In this case it was assumed that the aneurysm was the origin of recurrent embolisms. Besides those patients with previous aneurysmal bleeding, the aneurysm was found incidentally in asymptomatic patients by neuroradiology examinations in 4/28 patients (14%, see Figure 2); four patients (14%) had unspecific symptoms like headache or dizziness. One patient presented with symptoms indicative of frontal lobe dysfunction caused by a giant aneurysm in the supraophthalmic ICA.  Table 1 summarizes the clinical data.

The aneurysm size was classified as small (<10 mm; *n* = 15), large (10–20 mm; *n* = 11), and giant (>20 mm; *n* = 2). In 39% (11/28 patients), the diameter of the neck was <5 mm, in 43% the diameter was 5–10 mm (12/28 patients), and in 18% the diameter was >10 mm (5/28 patients). The configuration appeared in 19 cases wide-necked (68%) and in 7 cases fusiform (25%). The aneurysms were mainly located in the anterior carotid circulation (71%; 20/28). Most of them were detected in the internal carotid artery (ICA = 19; 68%) and one was detected in the medial cerebral artery (MCA = 1; 4%). Four aneurysms were located in the basilar artery (14%) and four in one of the vertebral arteries (14%).

### 2.2. Endovascular Treatment and Stent Data

All procedures were performed under general anaesthesia. All cases were discussed in an interdisciplinary conference. The indication was established in more complex, formerly untreated aneurysms (especially wide-neck, giant, and fusiform) or pretreated reperfused ones.

For the endovascular treatment of aneurysms by flow diversion we used two different high profile stents. For 23/28 patients we chose the SILK flow diverter (SFD, Balt Extrusion, Montmorency, France). We implanted two SFDs in a telescoping way in 2 patients (25 SFDs were placed in 23 patients). The SFD is a flexible, self-expanding device specifically designed to produce a hemodynamic flow diversion and to reconstruct laminar flow in the parent artery. The device is a braided mesh cylinder with flared ends, composed of 48 nickel-titanium (nitinol) alloy and platinum microfilaments of approximately 35 *μ*m. It is designed to provide 35% to 55% metal coverage of the target vessel's inner surface with a pore size of 110 to 250 *μ*m at nominal diameter [9].

In 5/28 cases the Pipeline Embolization Device (PED, Covidien, Mansfield, Massachusetts) was chosen for the reconstruction of the vessel wall. We placed 9 PEDs in 5 patients; four of them got 2 PEDs. The PED is a composite braided mesh tube of 48 strands with 75% cobalt chromium and 25% platinum. The diameter of the single wire is 30 *μ*m. At the nominal diameter, the pore size is 0.02–0.05 mm^2^ and the radial force is about 2.0 mN/mm (3.0 mm vessel diameter), which is similar to a SFD. The braided wires are loose on both ends. The PED is preinstalled on a stainless steel wire and is attached distal to a capture coil. A radiopaque 15 mm platinum tip extends beyond the end of the PED [4].

The diameter of the high profile FD employed ranged in size within 2.5–5.5 mm and in length within 15–50 mm.

In 7 procedures side branches of the aneurysm-sustaining artery were covered by the device (3 in the basilar artery).

In 23 cases (82%), implantation of the FD was placed as the first treatment. The remaining 5 cases received the implantation of a FD as a retreatment after clipping of another aneurysm (*n* = 2; 7%), another case after clipping and stent-supported coiling of the same aneurysm (*n* = 1; 4%), and again another case after stent-supported coiling of the same aneurysm (*n* = 1, 4%). In one woman a FD was implanted in another center. Four of those patients, who were pretreated, had previous SAH. Therefore we implanted FD as the first treatment in the parent artery of ruptured aneurysms in 3 cases.

### 2.3. Drug Management

All patients received dual antiplatelet therapy (aspirin 100 mg/d and clopidogrel 75 mg/d) from 2 to 28 days before procedure (mean: 12.9 days). One woman received prasugrel instead of clopidogrel because of previous implantation of a drug-eluting stent in a coronary artery. In this case we did not change medication. If the period to procedure was less than 3 days, a loading dose of 500 mg aspirin and 300 mg clopidogrel was applied. Responder status for aspirin and clopidogrel was detected before treatment (8% nonresponder aspirin; 16% nonresponder clopidogrel). For this purpose the assay for the quantitative in vitro determination of platelet function triggered by TRAP (thrombin receptor activating peptide) was used. Cut-off level for aspirin nonresponder was TRAP test > 60 U and aspirin > 70 U and for clopidogrel nonresponder was TRAP test > 60 U and clopidogrel > 45 U. To prevent the peri-interventional formation of a thrombus, heparin was provided controlled by antiplatelet clotting time (ACT). The therapeutic protocol after treatment in the case of normal responders was as follows: dual antiplatelet therapy for three months (aspirin 100 mg/d and clopidogrel 75 mg/d); after three months clopidogrel was stopped while aspirin 100 mg/d was continued as a life-long regime. In patients who did not sufficiently respond to aspirin or clopidogrel, we applied double-dose and repeated check of response in the course.

### 2.4. Follow-Up Schedule

In the course of the observation period, 26/28 patients underwent DSA at least once (most of them 3 months after treatment). One patient moved abroad and was therefore lost to follow-up by DSA. Another patient with multiple myeloma got MR imaging instead of DSA to prevent kidney dysfunction. A third patient was lost to follow-up 6 months after the procedure due to intracerebral bleeding (ICB) with lethal effect during a second FD session. Computed Tomography Angiography (CTA; *n* = 22), Magnetic Resonance Imaging (MRI; *n* = 2), or Digital Subtraction Angiography (DSA; *n* = 4) was performed in all patients during their hospital stay within three days after treatment. Follow-up was carried out by DSA, CTA, or MRI on 6 weeks, 3 months, 6 months, 1 year, 2 years, and 3 years. Angiographic outcome of treated aneurysms was assessed using a simplified type of the O'Kelly-Marotta grading scale (OKM), which classifies aneurysm based on angiographic filling and stasis of contrast material exclusively in the arterial phase in a four-level scale (A-D) as follows: A—complete (>95%); B—incomplete (5–95%); C—neck remnant (<5%), or D—no filling (0%) [10]. The clinical treatment effect was rated on a four-level scale (1—improvement, 2—delayed improvement, 3—no improvement, and 4—fatal outcome) and was not applicable (n.a.) in asymptomatic patients with incidental aneurysm.

## 3. Results

### 3.1. Technical Findings in Stent Deployment and Additional Appliance

The FD could be placed in a proper position across the parent artery of the aneurysm in 19/28 patients. Stent size was changed in one case to ensure complete unfolding. Due to suboptimal opening in a circumscribed part of the FD, in 6 patients additional balloon-dilatation was performed in an attempt to gain better results. In one of these patients (broad-based aneurysm arising from a fenestration located at the confluence of both vertebral arteries), additional stenting was performed to achieve better aneurysm occlusion. In 3 patients, a second FD, placed in a telescopic way during the on-going session, was necessary for sufficient haemodynamic exclusion of the aneurysm sack.

During two procedures, thrombotic material accumulated at the inner surface of the stent. In both cases abciximab (Reopro®, Janssen Biologics, Netherlands) was applied locally with resolution of the thrombotic material, followed by a continuous infusion of a maintenance dose for 12 h.

### 3.2. Postprocedural Neurological Events

During the early postprocedural period, 4/28 (14%) patients had neurological deficits related to the procedure. One patient (patient number 6) with a complex proximal basilar artery aneurysm and peri-interventional thrombotic clots requiring application of abciximab developed dysarthria and paresis of the right arm. MR imaging two days following the procedure demonstrated ischemic lesions on both sides of the cerebellum and within the thalamus and splenium of the left side. One patient (patient number 26) who presented initially with a ruptured aneurysm remained intubated because of recurrent nasopharyngeal bleeding under combined heparin and antiplatelet therapy. At the time of transfer to another center, neurological symptoms including ptosis and anisocoria were still present as a result of the SAH. In another case (patient number 14), aphasia occurred after intervention during the hospital stay with complete resolution of the symptoms at discharge. In correlation to this symptom, CTA showed a circumscribed intraparenchymal bleeding of 15 mm diameter in the dorsal temporal lobe of the left hemisphere with treated ICA aneurysm on the right side. Patient number 3 showed transient psychotic symptoms including agitation and loss of time orientation. No early or delayed aneurysm rupture occurred.

A complete recovery of headache related to the aneurysm was observed in one patient within the postprocedural hospital stay; an improvement was observed in further 6 patients with previous restriction of cranial nerve function. In those 6 patients a regression of ptosis, diplopic images, hemianopsia, and an improvement of vision were observed. The symptoms have been preexisting for 9 days to 6 months till the deployment of a FD.

### 3.3. Radiological Follow-Up and Aneurysm Occlusion

Due to the flow diverting effect of the high profile stent and thus the reduction of the blood flow into the aneurysm, we witnessed initiating thrombosis in 2/28 patients (7%) already at the end of procedure. In another 9 patients (32%) a delayed inflow and contrast material stagnation inside the aneurysm lumen was observed periprocedurally. Brain imaging by MRA and/or CTA performed within 2-3 days after deployment of a FD showed complete aneurysm occlusion in 4 of 28 cases (14%). One of those had been treated with additional coil packing at the same session and another one a second FD.

After discharge, during a follow-up range from 6 weeks to 3 years, control-imaging studies were available for 27/28 patients (after 6 months 26/28) at different time intervals. 3 months after procedure we observed complete aneurysm occlusion in 16 of 27 patients (59%). Six months after stent implantation, 63% (17/27) of the aneurysms were eliminated, a proportion that increased to 73% (19/26) after 1 year and 77% (20/26) two years after intervention.

When we applied the OKM, we evaluated the occlusion rate only of those patients who underwent DSA because the grading scale is based on recognizable angiographic characteristics [10]. During the course of the observation period, 26 of 28 patients got DSA imaging. The distribution of the different OKM degrees is shown in Table 2.

77% (20/26) achieved best results according to OKM grade D (no filling). In 14 of those 20 cases (70%) we observed best results with no contrast material filling of the aneurysm 3 months after FD deployment, 90% after 1 year and 100% after 2 years (see Table 3).

Most of the patients (22/26) underwent control imaging by DSA till 3 months after procedure. At this time, besides 14 patients with grade D, 6 of 22 patients (27%) had grade C (entry remnant) and 2 patients (9%) had grade B (subtotal filling). None of them had grade A (total filling). During follow-up we did not detect any aneurysm recurrence or progress in aneurysm filling.

A vessel originating from the aneurysm or the base of the aneurysm was evident in 18% (5/28 patients). Three (patients numbers 9, 20, and 22) achieved OKM grade D within the 3 months' follow-up, one patient (patient number 19) achieved OKM grade B after 3 months, and one patient (patient number 2) achieved OKM grade D after 2 years. However, due to the small number, no statistically significant results of this subgroup can be stated.

In 4 of 6 patients who did not achieve grade D (except one who have not achieved best result until 2 years after FD deployment), proximal and/or distal stent overlapping ≥5 mm was not guaranteed sufficiently.

### 3.4. Neurological Results and Complications during Follow-Up

In addition, the clinical-neurological outcome following stent implantation was evaluated. 8/17 patients (47%) with former focal symptoms or cranial nerve palsy were rated as clinically improved (level 1). None of them had to be retreated and no recurrence was seen. We ranked 5/17 patients (29%) among category 2 (delayed improvement); in three cases (18%) no regression of former focal symptoms was seen (treatment effect level 3). In one of those cases (patient number 12) even worsening visual impairment was observed. The period between the appearances of the first symptoms until treatment was 6 months. Concerning another patient (patient number 21) the damage of the abducens nerve by prolonged compression preceded the intervention for 1.5 years. In this patient no recovery was observed. The third patient (patient number 15) with level 3 presented with a giant aneurysm (max. diameter 22 mm) with a strong jet-like flow. We decided to implant two FDs in a telescoped way to ensure the reconstruction of the parent artery. This patient was lost to follow-up. That is why time lacked to improve. Consequently we had to rank this patient in level 3. In one patient (patient number 19) an ICB in the contralateral hemisphere occurred shortly after the second FD session with lethal effect (treatment effect level 4).

The most frequent complication during follow-up (4/27; 15%) was in-stent stenosis (ISS, see Figure 2). The majority of the stenoses (3 of 4) had no hemodynamic or clinical relevance and showed a regression in the degree of stenosis during follow-up. In only one case of ISS (patient number 7), additional percutaneous transluminal angioplasty (PTA) was performed (balloon-dilatation 5 months after FD placement and stenting 7 months after FD placement) due to high grade of stenosis. Being initial clopidogrel nonresponder, this patient took clopidogrel 150 mg/d for 1.5 years. After reperfusion of the aneurysm, clopidogrel was reduced. Two years after treatment the aneurysm occluded and clopidogrel was stopped. In the remaining 3 patients with ISS no PTA was needed but we continued clopidogrel therapy with a higher dose (150 mg/d). The 3 remaining stenosis cases fortunately achieved OKM grade D 3 months after FD placement and had no subsequent reperfusion. During follow-up we observed vascular obliteration in 2 patients (7%). In one case the anterior inferior cerebellar artery (AICA) occluded which originated from the aneurysm basis with complete leptomeningeal collateralisation after one year (patient number 22). In the other case a segmental obliteration of the left vertebral artery (VA) on a level with the parent artery of the aneurysm occurred (patient number 28). In both cases the patients did not show any clinical-neurological abnormalities. A second FD was placed in one of two patients (patient numbers 14 and 17) who developed an endoleak 3 to 9 months after treatment. OKM grade D could be reached 6 months after former FD deployment. No delayed SAH was observed during follow-up. Complications during follow-up are summarized in Table 4.

A supplementary table providing all patients' clinical symptoms, aneurysm size, and location as well as treatment effects is provided (see supplementary materials).

## 4. Discussion

The primary goals of aneurysm treatment should be a complete occlusion of the aneurysm sack to prevent delayed SAH or recurrence of the aneurysm lumen as well as recovery from neurologic symptoms. However, it is hard to define the optimal management, especially dealing with incidental finding on an imaging study undertaken for another purpose. The dilemma arises whether the risk associated with preventive surgical or endovascular treatment is outweighed by the risk of death or disability from rupture of the untreated aneurysm. Korja et al. [11] report that the lifelong risk of an unruptured intracranial aneurysm rupture depends strongly on the risk factors, also other than the size and location (e.g., cigarette smoking, sex, and systolic blood pressure), and these should be taken into account when making treatment decisions. Another study details that the rupture risk of growing unruptured cerebral aneurysms is 5% in 5 years. They identified additional risk factors like lobulated configuration, multiple aneurysms, and growth [12].

The International Subarachnoid Aneurysm Trial (ISAT) and other recently published data confirm and reinforce their preliminary findings that rebleeding was more likely after endovascular coiling than after neurosurgical clipping, but the risk was small and the probability of disability-free survival was significantly greater in the endovascular group than in the neurosurgical group at 10 years [13, 14].

To improve occlusion rate and to overcome the faintness of coil compaction, aneurysm recurrence, and rebleeding after coiling, a paradigm shift in interventions from an endosaccular to an endoluminal reconstruction was promoted by the design of the FD. The aneurysm itself does no longer have to be catheterized. Comparison of flow diversion and coiling of large unruptured aneurysms shows a significantly higher proportion of aneurysms treated with PED with complete obliteration and fewer necessities of retreatment compared with coiled aneurysms [15].

Meanwhile, the FD asserts itself not only in the treatment of large saccular aneurysms but also in wide-necked and fusiform ones or aneurysms that have a branch incorporated into the sac. The latter, flow through the FD, is maintained into the branch originating from the sac while occlusion of the aneurysm is still possible [2]. Our results do not deviate from this mechanism, except one occlusion of the AICA in a patient showing preexisting extreme atherosclerosis and without clinical relevance in the follow-up. Referring to this, another survey, dealing with SFD implantation in the basilar artery, distinguishes between occlusion of a side branch shortly after FD implantation by narrowing the orifice mechanically or blocking the orifice by tiny thrombi formed on the surface of the FD and late infarcts caused by neointimal overgrowth and progressive narrowing of the perforator's orifice [9]. The mechanism that keeps the side branches and perforators open might be the blood downstream, following the pressure gradient to the low-pressure tissue. The fact that the supplied area acts as a consumer might contribute to the maintenance of sufficient perfusion. Both cases of vessel occlusion in our series (AICA and segment of VA) appeared in patients with extreme atherosclerotic transformation, which has to be considered when making treatment decisions.

In our series, the aneurysmal occlusion rate turns out to be 59% after 3 months, increasing to 77% due to implantation of a second FD or deviation of the antiplatelet regime. Subtotal occlusion was obtained in further 15%. Summing up, 92% of the patients achieved best or good results concerning occlusion rate. It seems to be controversial that a small neck remnant at follow-up is often accepted as adequate treatment after conventional aneurysm coiling, whereas slight filling of an aneurysm treated with a FD may be enough to perpetuate continued mass effect, progressive aneurysm growth, and in some cases spontaneous rupture [10]. According to the OKM grading scale 77% achieved grade D, 70% of them already 3 months after procedure and 80% after 6 months. These results go along with data from a meta-analysis where complete occlusion rate was 76% (95% CI, 70%–81%) at 6 months [1]. The rate and time of complete occlusion were not correlated with the aneurysm size as in other retrospective studies [4, 16–18].

Analysing the characteristics of aneurysm thrombosis, we found out that broad stent overlapping of the aneurysm orifice (≥5 mm proximal and distal) is an important predictor of early aneurysm occlusion. In 4 of 6 patients who did not achieve grade D (except one who has not achieved best result until 2 years after FD deployment), proximal and/or distal stent overlapping ≥5 mm was not guaranteed sufficiently. This kind of device deformation was also described in a clinical case report supplemented by in vitro studies [19]. Estrade et al. [19] tried to identify the causes and effects of device deformation. The main finding of this report is that, upon deployment of oversized FDs, landing zones of insufficient length may lead to device deformation with terminal stenosis. Even though the stenosis may not be flow limiting, the inadequate apposition of the device to the wall of the parent vessel may cause thromboembolic complications. Implanting an oversized FD sometimes cannot be avoided because of diameter alteration between proximal and distal parent artery of the aneurysm. The highest coverage (and therefore least porosity) is achieved at full expansion of the device. Particularly the transitional zone between the constrained part and the fully opened part shows reduced coverage when the oversized device opens to its nominal size within the aneurysm [19, 20]. A residual more turbulent inflow into the aneurysm sac may result that can explain failures and recurrences [19]. More recent generation of ED devices with higher radial forces and tapered design may further improve occlusion rates by allowing correct stent sizing despite diverging parent vessel diameters. Consequently the selection of the adequate length and diameter of the device to guarantee a proper stent adhesion to the vessel wall remains important. As shown in haemodynamic analysis of aneurysm occlusion by flow diversion in rabbits, imperfect deployments can have a substantial impact on the occlusion time and outcome of flow diverting procedures [18].

It is important to mention that 2 of the 6 patients who did not achieve best results in aneurysm occlusion had a situation with 2 branching vessels jailed, which might be conducive to delayed aneurysm exclusion from blood flow. The presence of incorporated vessels has already been reported causing delayed occlusion at 6 months' follow-up and 1 year' follow-up, but not at 2+ years in a recent published study of Chiu et al. [16], which is in concordance with our experience. 2/5 patients in this subgroup did not achieve OKM grade D within 3 months.

The therapeutic effect of the endovascular treatment by flow diversion became most obvious by looking at the most common aneurysm related symptom, the cranial nerve palsy (12/28 patients; 43%). It becomes clear that the patients with visual loss, perimetric restriction, or palsy of the inner or outer ocular muscles achieved better results when treated by FD within 3 months after principal appearance of the symptoms. The time of their total or partial recovery ranged from 3 days to 9 months after procedure. Progressive aneurysm thrombosis and adjacent oedema after treatment might even cause initial worsening of neurological symptoms. Particularly pulsation of the aneurysm wall and adjacent oedema after treatment are responsible for the cranial nerve palsy [21]. If time range between first symptoms and FD implantation exceeds 3 months, the patients have a very restricted chance of recovery so that impairment of the inner or outer ocular muscles or a perimetric or visual restriction remains. Long period till treatment can be an indicator for recovery failure, because of gradually occurring partial calcification of the aneurysm wall and thus less potential of significant reduction in size and simultaneous reduction in mass effect and pulsation leading to cranial nerve decompression. The FD actually should surpass coiling in this feature. That is why treatment should be as prompt as possible after occurrence of first symptoms.

As no ischemic events or aneurysm rupture during follow-up occurred among the patients we treated, we cannot confirm the declaration that SFD is more likely to cause these delayed device-related complications than PED [22]. But, aside from the lower number of patients we treated with PED, we experienced the ISS to be the most frequent complication (4/27 patients; 15%). All of the patients who developed ISS were clinically asymptomatic, except one patient with an unspecific symptom like headache. In asymptomatic patients the angiographic finding was managed medically and with serial follow-up imaging. Additional PTA was performed in only one case. None of the patients with ISS underwent additional balloon-dilatation during their first FD session, which repels the idea that parent artery injury could be a trigger for neointimal proliferation. Analysing the data, we found neither any other correlation between the developments of ISS and other potentially relating factors, such as cardiovascular risk factors, nor lack of response to antiplatelet therapy. Despite the fact that the ingrowth of tissue over the stented segment very likely promotes the durability of blood flow redirection and aneurysm occlusion, occasionally the stenosis may result in neurological symptoms that require retreatment to restore flow. Although the majority of patients with ISS are asymptomatic, the induction of a symptomatic intracranial stenosis in this previously asymptomatic patient population represents a considerable issue [23].

Furthermore, it seems to be challenging to find a balance between aneurysm occlusion and prevention of ISS by varying in dose and period of antiplatelet therapy while facing the additional problem with therapy of nonresponder. A recently published study found a wide variability in the initial patient response to the standard 75 mg daily clopidogrel dose. They suggest that an “acceptable” 60–240 P2Y12 reaction units (PRU) range would not lead to increased thromboembolic or hemorrhagic complications in a larger patient population, but further studies are needed to ensure this statement [24].

The antiplatelet medication and additional anticoagulation during procedure might be responsible for the fatal outcome of an ICB on the contralateral hemisphere of the target aneurysm during a subsequently performed FD procedure in one of our patients (4%). This result equals the rate of Brinjikji's et al. [1] meta-analysis regarding procedure-related mortality of 4% (95% CI, 3%–6%) and is comparable with their ICB rate of 3% (95% CI, 2%–4%), with 3% experiencing early ICB and 2% experiencing late ICB. Aneurysm size and location were not significantly associated with ICB rate. The indication for a second FD in our patient was provided with persistent perfusion of the aneurysm despite termination of dual antiplatelet therapy and proceeding deprivation of vision. The complication became noticeable after delayed awakening and was confirmed by CT. Other cases of ICB are reported. Unlike our case report, all haemorrhages were anatomically remote from the treated aneurysm, but the hematomas were situated in the ipsilateral cerebral hemisphere. The occurrence of ICB seems to be more frequent than delayed aneurysm rupture (1% in the RADAR survey [25]). Therefore ICB has recently emerged as a threatening complication of intracranial aneurysm treatment with FDs. While the mechanism and related factors of ICB during FD procedure remain poorly understood, several considerations could be proposed to explain the occurrence of delayed ipsilateral ICB after flow diversion procedure. Haemodynamic alteration from flow diverter placement, haemorrhagic transformation of ischemic stroke, dual antiplatelet therapy, and cardiovascular risk factors are presumed mechanisms [26].

## 5. Conclusion

In conclusion, endovascular reconstruction using a FD represents an effective and safe treatment in those aneurysms that are not suitable for conventional interventional or surgical treatment. In giant, wide-necked, and fusiform aneurysms, the occlusion rate is encouraging and in our series no recurrence or delayed SAH occurred. Nevertheless, we found a noteworthy incidence of in-stent stenosis (ISS, 4/27 patients; 15%), but fortunately among those the incidence of symptomatic ISS or the necessity of retreatment is very low (1/4). Another complication that comes along with increased thrombogenicity is the occlusion of the parent artery, especially in very atherosclerotic vessels with clinical relevance depending on the degree of collateralisation. The heterogeneity of the response [25] to clopidogrel could explain the ISS on one hand and the haemorrhagic events on the other and has to be considered strictly.

However, the complexity of the described issues underlines the necessity of an interdisciplinary consensus concerning treatment in this cohort.

## Supplementary Material

Supplementary Table 1: The table shows patients' characteristics of all 28 patients including age, sex, clinical symptoms, aneurysm size and location, special findings, treatment effect, and clinical outcome in the follow-up period.

## Figures and Tables

**Table 1:** Clinical data.

Aneurysm location		Aneurysm size (mm)		Clinical symptoms		Configuration	
Anterior circulation	20	<10	15	SAH	7	Wide-necked	19
BA	4	10–20	11	Incidentally	4	Fusiform	7
VA	4	>20	2	Focal symptoms (*cranial nerve palsy*)	17 (*12*)	other	2

This table summarizes location, size, and configuration of aneurysms as well as the clinical symptoms of the patient cohort. BA: basilar artery; SAH: subarachnoid hemorrhage; VA: vertebral artery.

**Table 2:** Best results according to O'Kelly-Marotta grading scale.

	Frequency	%
A	1	4
B	1	4
C	4	15
D	20	77
Total amount	26	100

This table shows the follow-up results of digital subtraction angiography (DSA) in 26 patients (in 2 patients only CTA or MRI was performed). OKM: A total filling, B subtotal filling, C entry remnant, and D no filling.

**Table 3:** Point in time (months) of OKM grade D (no filling).

Months	Frequency	Cumulative %
3	14	70
6	2	80
12	2	90
24	2	100
Total amount	20	100

Point in time (months) of the 20 patients who were rated as OKM grade D (no aneurysm filling).

**Table 4:** Complications during follow-up.

	Frequency	%
None	18	67
Endoleak	2	7
Stenosis	4	15
Vascular obliteration	2	7
ICB	1	4
Total amount	27	100

This table summarizes complications during follow-up. There was no need for subsequent intervention except one in-stent stenosis and one endoleak. One patient with intracerebral bleeding (ICB) during the second FD session died.
