# Supplementary material for: Morphologic and Clinical Outcome of Intracranial Aneurysms after Treatment Using Flow Diverter Devices: Mid-Term Follow-Up
Source: Radiol Res Pract. 2016 Feb 23;2016:2187275. doi: 10.1155/2016/2187275 (PMC4781985; doi:10.1155/2016/2187275)
Supplement: Supplementary file 1 — Supplementary Table 1: The table shows patients' characteristics of all 28 patients including age, sex, clinical symptoms, aneurysm size and location, special findings, treatment effect, and clinical outcome in the follow-up period. [file 2187275.f1.pdf]

Supplementary material showing patients´ characteristics and results in detail

Pat. No.	Age	Sex	Initial Symptoms	Aneurysma (location, diameter mm)	Specifics	Complications	OKM	Treatment Effects
1	55	f	Left oculomotor nerve palsy	ICA left, 15	none	none	D	1
2	8	m	Headache	BA, 26	IV	none	D	2
3	39	m	Dizziness, headache	VA left, 11	none	none	**D**	1
4	51	f	SAH	BA, 20	Recurrence after stent-assisted coiling	none	C	2
5	75	f	Right abducens nerve plasy	ICA right, 13	none	none	**D**	1
6	54	f	Incidental finding	BA, 8	Coiling, PTA, Reopro®	Cerebellar Ischemia	**D**	n.a.
7	59	f	Left oculomotor nerve palsy	ICA left, 19	PTA, Stent in the course	Stenosis	C	2
8	39	f	SAH	ICA left, 3	Recurrence after clipping and stent-assisted coiling	none	C	2
9	43	m	TIA	ICA left, 4	PTA, Reopro®, IV	none	**D**	1
10	62	f	Right oculomotor nerve palsy	ICA right, 12	none	none	**D**	1
11	61	f	Incidental finding	MCA right, 8	none	Stenosis	**D**	n.a.
12	60	f	Optical nerve palsy	ICA left, 21	2 FDs during the same session	none	n.a.	3
13	52	f	Right sensual trigeminal nerve palsy	ICA right, 23	none	none	**D**	1
14	61	f	Right optic and oculomotor nerve palsy	ICA right, 16	PTA, Second FD in the course	Endoleak	D	2
15	56	f	Right optic and oculomotor nerve palsy	ICA right, 22	2 FDs during the same session	none	n.a.	3
16	52	m	SAH	VA right, 3	2 FDs during the same session	none	D	1
17	60	f	Left oculomotor and abducens nerve palsy	ICA left, 11	none	Endoleak	D	2
18	52	f	SAH	ICA left, 4	none	none	**D**	1
19	54	f	Left optic and oculomotor nerve palsy	ICA left, 12	Second FD in the course, IV	ICB right during second FD session	B	4
20	54	f	Frontal lobe disorder	ICA right, 16	IV	none	**D**	1
21	83	f	Optic and left abducens nerve palsy	ICA left, 16	PTA	none	A	3
22	55	m	SAH	BA, 6	IV	Vascular obliteration	**D**	1
23	50	f	SAH	ICA left, 4	Second FD in the course	none	**D**	1
24	22	f	Left glossopharyngeal nerve palsy	ICA left, 16	none	none	**D**	1
25	44	m	Incidental finding	ICA left, 5	none	Stenosis	**D**	n.a.
26	63	f	SAH after FD ex domo	ICA right, 6	PTA	Stenosis	**D**	2
27	71	f	Dizziness	VA right, 11	PTA	none	C	2
28	70	f	Incidental finding	VA left, 5	none	Vascular obliteration	**D**	n.a.

The supplementary table shows all patients including clinical symptoms, aneurysm size, and location as well as treatment effects. We used the O'Kelly-Marotta grading scale (OKM), which classifies aneurysm filling in: A – complete (>95%); B – incomplete (5-95%); C – neck remnant (<5%) or D – no filling (0%), the bold character **D** was used for OKM D within the first 3 months after treatment. OKM was not applicable (n.a.) in two patients who did not undergo digital subtraction angiography in the follow-up. The clinical treatment effect was rated on a four level scale (1 – improvement, 2 – delayed improvement, 3 – no improvement, 4 – fatal outcome) and was not applicable (n.a.) in asymptomatic patients with incidental aneurysm. BA – basilar artery, ICA – internal carotid artery, MCA – medial carotid artery, PTA – percutaneous transluminal angioplasty, SAH – subarachnoid hemorrhage, TIA – transient ischemic attack, VA – vertebral artey, IV – incorporated vessel
